# Deoxycholic acid aggravates necrotizing enterocolitis through downregulation of mesenchymal-epithelial transition factor expression

**DOI:** 10.1590/1414-431X2024e14046

**Published:** 2024-12-02

**Authors:** Hongfu Li, Jiahao Lai, Dongfan Xiao, Dabin Huang, Yinchun Zhang, Xia Gu, Fei Li, Hu Hao

**Affiliations:** 1 Department of Pediatrics, The Sixth Affiliated Hospital, Sun Yat-sen University, Guangzhou, China; 2 Department of Respiratory and Critical Care Medicine, Affiliated Hospital of Guangdong Medical University, Zhanjiang, China; 3 Biomedical Innovation Center, The Sixth Affiliated Hospital, Sun Yat-sen University, Guangzhou, China; 4 Inborn Errors of Metabolism Laboratory, The Sixth Affiliated Hospital, Sun Yat-sen University, Guangzhou, China

**Keywords:** Neonatal necrotizing enterocolitis, Infant, Newborn, Intestinal epithelial cell, Deoxycholic acid

## Abstract

Bile acids are closely associated with necrotizing enterocolitis (NEC), and their accumulation has cytotoxic effects on cells. However, the specific bile acid subtype involved in NEC and its underlying mechanisms remains poorly understood, limiting the therapeutic potential of bile acids as treatment targets. In the present study, deoxycholic acid (DCA) accumulation in the intestinal lumen exacerbated NEC-induced intestinal damage. DCA suppressed the expression of mesenchymal-epithelial transition factor (MET), a proto-oncogene located on chromosome 7q31.2 that encodes c-Met, in the mouse intestine through transcription factors and increased nuclear translocation of p-STAT3. MET is a receptor tyrosine kinase that participates in cell proliferation and migration processes. Increasing concentrations of DCA downregulated MET expression and reduced the proliferation and migration of intestinal epithelial cells *in vitro*. MET knockdown reduced the proliferation and migration of intestinal epithelial cells but increased STAT3 phosphorylation. These findings indicated that MET mediated STAT3 involvement in intestinal epithelial cell proliferation and migration, demonstrating that the inhibitory effect of DCA on MET disrupted this process. These results elucidated the damaging effects and mechanisms of DCA accumulation in NEC, providing new insights into the use of DCA as a therapeutic target for NEC.

## Introduction

Necrotizing enterocolitis (NEC) is one of the major diseases leading to mortality in premature infants, with an incidence rate of 10% and a mortality rate as high as 50% among very low birth weight infants (1). NEC primarily affects the distal ileum and colon, and it is characterized by inflammatory changes, mucosal sloughing, villous atrophy, and, in severe cases, necrosis and perforation of the intestinal wall (2). Surgical resection of necrotic intestinal tissue is currently the preferred treatment for severe NEC, but it often leads to complications, such as short bowel syndrome and intestinal failure (3). Survivors of NEC surgery also face a high risk of neurodevelopmental sequelae (4). The lack of curative treatment methods underscores the incomplete understanding of the pathogenesis of NEC, highlighting the urgent need for further research in this area.

Intestinal epithelial cells play crucial roles in nutrient absorption and the repair of intestinal injuries (5). Prematurity, hypoxic-ischemic injury, infection, immune dysregulation, dysbiosis, and improper feeding are recognized risk factors for NEC (1). Increasing evidence suggests that abnormalities in bile acid metabolism may contribute to NEC (6-8). Our previous studies have revealed significant alterations in bile acid levels in the peripheral blood of NEC patients (9). Other researchers have shown that variations in fecal bile acid levels and secondary bile acid levels in the peripheral blood are potential biomarkers of NEC (10,11). These findings suggest a close association between bile acid levels and NEC. The distal ileum is an important site for the enterohepatic circulation of bile acids, and inflammation in this region can lead to bile acid retention in the intestine (12,13).

The effect of secondary bile acids is complex. While hydrophobic secondary bile acids, such as DCA and lithocholic acid (LCA), are cytotoxic, hydrophilic secondary bile acids, such as ursodeoxycholic acid (UDCA), have a protective effect (14,15). One of the ways in which UDCA protects the intestine is by inhibiting DCA-induced apoptosis (16). These findings indicate that DCA may act as an injurious factor in NEC. Elevated serum DCA levels have been observed in NEC patients, and DCA accumulation has been implicated in intestinal epithelial cell damage and inflammation (11,17). Our preliminary studies have demonstrated that increased DCA concentrations impair intestinal organoid viability (18). These results suggest that the occurrence of NEC may lead to the retention of DCA and subsequent intestinal injury. Therefore, DCA may represent a potential therapeutic target for treating NEC.

Mesenchymal-epithelial transition factor (MET) is a membrane receptor tyrosine kinase that, upon binding to its ligand HGF, activates pathways such as AKT and STAT3, which are involved in cellular processes like proliferation and migration. In studies of skin injury, MET-knockout mice exhibit impaired wound healing, with only residual MET-expressing epithelial cells capable of completing the repair process (19), which involves the regulation of cell adhesion and the cytoskeleton. Specifically, MET regulates the expression of the small G protein Arf6 mRNA in response to injury, facilitating repair (20). In the intestine, MET promotes epithelial cell migration by loosening tight junctions upon ligand binding (21). Additionally, MET expression is associated with epithelial cell proliferation during intestinal injury induced by mucosal inflammation of the small intestine. These findings suggest that MET may play a crucial role in the repair of intestinal epithelial cells in NEC.

Given that DCA accumulation has been shown to affect protein expression in intestinal epithelial cells, we hypothesize that NEC-induced DCA accumulation at high concentrations could impair intestinal repair function by altering protein expression. In this study, we investigated the impact of NEC on bile acid levels in mice and analyzed transcriptional changes in intestinal epithelial cells from NEC patients. We also examined the effects of DCA on protein expression in intestinal epithelial cells and its role in injury in animal models of NEC.

## Material and Methods

### Construction of the NEC mouse model and animal grouping

Six-week-old mice were purchased from the Ruige Biological Technology Co., Ltd. (China) and bred at the Animal Center of Seyotin Biological Technology Co., Ltd. (China). All animal experiments were approved by the Ethics Review Board of Seyotin Biological Technology Company. In accordance with previously established methods (18), 10-day-old pups were used to establish the model. The pups were administered a formula milk mixture consisting of Similac Advance infant formula (Abbott Nutrition, USA) and Esbilac milk replacement for puppies, (PetAg, USA) at a ratio of 2:1, with a dosage of 40 µL per gram of body weight. This mixture, supplemented with lipopolysaccharide (LPS, #L2880, Sigma, USA) at a concentration of 0.025 µg/µL, was administered via gavage five times daily. The animals were subjected to a regimen consisting of a 3-min exposure to 100% nitrogen followed by a 10-min cold (4°C) stimulation session, which was administered twice daily. To examine the impact of DCA (DCA, #ST2049, Beyotime, China) on NEC, various concentrations of DCA, including 0 mg/mL (NEC group), 2.5 mg/mL (DCA 2.5 group), and 5 mg/mL (DCA 5 group), were added to the formula milk.

### Cell culture

IEC-6 cells were cultured in DMEM (#C11995500BT, Thermo Fisher Scientific, USA) supplemented with 10% FBS (FSP500, ExCell Bio, China) at 37^o^C in a 5% CO_2_ incubator. When 70-80% confluence was reached, the cells were passaged using 0.25% trypsin with EDTA (#25200056, Thermo Fisher Scientific).

### Cell proliferation assay

DCA was initially dissolved in ddH_2_O to produce a stock solution of 200 mM, which was subsequently filtered through a 0.22-µm membrane. Prior to application, thorough dissolution of DCA was ensured, which was facilitated by mild heating in a water bath. Upon reaching 70-80% confluence, IEC6 cells were detached using trypsin, seeded onto 96-well plates at a density of 2000 cells per well, and maintained in 100 µL of medium per well. After 24 h, the cells were grouped based on the concentration of DCA in the medium as follows: 50, 100, 150, 200, and 250 µM. After removing the medium, 100 µL of medium containing the indicated concentration of DCA was added to each well. Following a 24-h incubation period, 10 µL of CCK8 working solution (Dojindo, Japan) was added to each well, followed by further incubation at 37°C for 4 h. The absorbance was then measured at 450 nm using an enzyme-linked immunosorbent assay reader (PerkinElmer, USA).

### Cell migration assay

Lines were drawn on the bottom of a 6-well plate as initial positioning references. Cells were seeded onto a 6-well plate and allowed to grow to 100% confluence. Straight scratches were made perpendicular to the reference lines using a pipette tip. The plate was washed twice with PBS to remove debris, and fresh medium or medium supplemented with 200 µM DCA was added. After 24 h, images were captured under a microscope (IX73, Olympus, Japan).

### MET knockdown assay

The pLKO.1-puro plasmid driven by the U6 promoter was used to construct the plasmid for MET knockdown, with the shRNA fragment inserted between the EcoRI (#R3101, NEB, USA) and AgeI (#R3552, NEB) sites. Subsequently, the plasmids, along with psPAX2 and pMD2, were co-transfected into 293T cells using Turbofect (#R0531, Thermo Fisher Scientific) according to the manufacturer's instructions, and the virus-containing supernatant was harvested after 48 h. The virus supernatant was then used to transduce IEC-6 cells using polybrene (#TR-1003, Sigma) according to the manufacturer's protocol, followed by selection with 5 µg/mL puromycin for 24 h. The sequences of the targeted MET shRNAs used were: sense, CCGGGCGGCAATTCTAGACACATTTCTCGAGAAATGTGTCTAGAATTGCCGCTTTTT; antisense, AATTAAAAAGCGGCAATTCTAGACACATTTCTCGAGAAATGTGTCTAGAATTGCCGC. The non-targeted negative control sequences were: sense, CCGGGCGGCAATTCTAGACACATTTCTCGAGTTCTCCGAACGTGTCACGTTTCTTTTT; antisense, AATTAAAAAGAAACGTGACACGTTCGGAGAACTCGAGAAATGTGTCTAGAATTGCCGC.

### Transcriptome sequencing and analysis

Intestinal tissue samples were collected from mice in the NEC and DCA5 groups and promptly frozen in liquid nitrogen. Total RNA extraction was performed using TRIzol (Invitrogen, USA) following the manufacturer's protocol. RNA quality was assessed using an Agilent 2100 Bioanalyzer (Agilent Technologies, USA), followed by RNase-free agarose gel electrophoresis. Following total RNA extraction and quality assessment, RNA samples that passed quality control were sent to Gene Denovo Biotechnology Co., Ltd. (China) for sequencing on the Illumina NovaSeq 6000 platform. The raw sequencing data were preprocessed using fastp (version 0.18.0) to eliminate reads containing adapters, reads containing more than 10% unknown nucleotides (N), and low-quality reads with a Q value ≤20. The paired clean reads were aligned to the reference genome using HISAT2.2.4, followed by RNA differential expression analysis between the two groups conducted using DESeq2 software in R. Differentially expressed genes (DEGs) were identified based on the following criteria: a false discovery rate (FDR) less than 0.05 and an absolute fold change ≥2. Subsequently, Kyoto Encyclopedia of Genes and Genomes (KEGG) pathway enrichment analysis of the DEGs was conducted utilizing the clusterProfiler package in R.

### Single-cell sequencing data analysis

The GSE178088 dataset was utilized for analysis. Sequence Read Archives (SRA) data were obtained and converted to fastq files using fasterq-dump software for subsequent analysis. Because the GSE178088 dataset is based on the 10X Genomics platform, the official “Cell Ranger” pipeline was used to align the data to the reference genome (GRCh38). To ensure data quality and reliability, quality control standards and tools referenced in the literature of the dataset were utilized. Quality control was performed using the “Seurat” package in R (22). The criteria for data removal were: 1) cells with mitochondrial read counts greater than 25% and 2) cells with gene counts fewer than 200 or greater than 7000. Clustering analysis was conducted on the transcriptome sequencing data of 17,267 cells (28,087 genes). PCA was employed to reduce the dimensionality of the high-dimensional single-cell sequencing data, which decreased the complexity of the data for visualization, understanding, and analysis. In the present study, the npc settings were set to 30. Through elbow plot analysis, “7” was selected as the dimension for subsequent analysis.

### Cell clustering

The FindNeighbors function in the Seurat package in R was used to calculate cell-to-cell similarity for subsequent clustering analysis, employing the first seven principal components. The FindClusters function in the Seurat package was utilized to identify cell clusters. After repeated testing, a resolution of 0.4 was used to identify cell clusters, and UMAP visualization was performed.

### Cell type identification

A strategy based on published literature was employed for identifying intestinal epithelial cells in single-cell sequencing data of intestinal tissue. First, epithelial cells were screened using the EPCAM epithelial cell marker gene. The intestinal epithelial cell clusters were further identified using the following intestinal epithelial cell marker genes: *ALPI*, *SLC26A3*, *TMEM37*, *FABP2*, *RBP2*, and *ANPEP* (23).

### Differentially expressed genes and enrichment analysis

The analysis of gene expression in the intestinal epithelial cell cluster was conducted using the FindMarkers function within the Seurat package. Differentially expressed genes were identified based on the criteria of P_val_adj <0.05 and |avg_log2FC| >1. These genes were then subjected to KEGG pathway enrichment and GO functional annotation analyses using the ClusterProfiler package, with statistical significance defined as P<0.05. Additionally, a protein-protein interaction (PPI) network for the differentially expressed genes was constructed using the STRING website, with the minimum required interaction score set to 0.400. The constructed PPI network was subsequently imported into CytoScape 3.9.1 software for Hub gene identification and visualization utilizing the CytoHubba module.

### Western blot analysis

Following collection, mouse intestinal tissues were ground in RIPA lysis buffer containing protease inhibitor and phosphatase inhibitor solution (RIPA, #P0013; protease inhibitor and phosphatase inhibitor solution, #P1045; Beyotime) according to the manufacturer's instructions. After 30 min of incubation on ice, the lysates were centrifuged at 13201 *g* for 20 min at 4°C. The supernatant was carefully transferred to a new centrifuge tube. Protein quantification was performed using the BCA method according to the manufacturer's instructions. Subsequently, the samples were boiled at 95°C for 10 min after the addition of 5× loading buffer (#P0015, Beyotime) and PBS (#10010031, Thermo Fisher Scientific). Protein electrophoresis was conducted using SDS-PAGE gels (#PG112, EpiZyme, USA), followed by transfer to nitrocellulose membranes. After the membrane was washed with tris-buffered saline with Tween 20 (TBST), the membrane was blocked with fresh 5% skim milk for 1.5 h. After three washes with TBST, the appropriate primary antibodies (MET, #25869-1-AP, Proteintech; Beta-actin, #20536-1-AP, Proteintech; p-STAT3, #9145, Cell Signaling Technology; STAT3, #9139, Cell Signaling Technology; all at 1:1000) were added and incubated overnight at 4°C. HRP-conjugated secondary antibodies (HRP-goat anti-rabbit recombinant secondary antibody, RGAR001, Proteintech; HRP-goat anti-mouse recombinant secondary antibody, RGAM001, Proteintech; both at 1:3000) were then added, followed by chemiluminescent detection. Quantification of target band intensities was performed using ImageJ (NIH, USA) densitometry software (v1.53 k).

### Injury evaluation

After routine H&E staining of the paraffin sections, scoring was performed according to the following pathological grading criteria (24): Grade 0, intact intestinal villi and epithelium with normal tissue structure; Grade 1, mild separation or swelling of the submucosa or lamina propria; Grade 2, moderate separation, swelling of the submucosa and/or lamina propria, with submucosal and/or muscular layer edema; Grade 3, severe separation of the submucosa and/or lamina propria, with submucosal and/or muscular layer edema, and localized villous loss; and Grade 4, disappearance of intestinal villi accompanied by intestinal necrosis. Scoring was conducted using a double-blinded method, with five fields of view assessed for each sample, and the average value was calculated as the final score. A score ≥2 was considered indicative of NEC onset (the criterion for successful modeling), with higher scores indicating more severe lesions.

### Immunofluorescence and immunohistochemistry

Paraffin sections were deparaffinized, and antigen retrieval was performed, followed by membrane permeabilization with 0.3% Triton-X100 for 10 min at room temperature. After washing with TBST, the sections were blocked with 5% BSA at room temperature for 1 h. Subsequently, the appropriate primary antibodies (MET, 1:200; p-STAT3, 1:400) were added and incubated overnight at 4°C. After three washes with TBST, the corresponding secondary antibodies were added at room temperature for 1 h, followed by three washes with TBST. For immunofluorescence, mounting medium containing DAPI was added for slide mounting and observation under an inverted fluorescence microscope (IX73, OLYMPUS, Japan). For immunohistochemistry, staining was performed using a DAB kit (#G1212, Servicebio, China), and the sections were counterstained with hematoxylin. Subsequently, neutral gum was added for slide mounting, and the slides were observed under a light microscope (DP27, Olympus).

### Bile acid detection

After mouse feces were obtained, the samples were freeze-dried. Mouse blood was centrifuged at 5000 *g* for 15 min at 4°C, and the upper layer of serum was collected. The samples were sent to Aidialife Company (China) for detection.

### Statistical methods

Normally distributed data are reported as means±SD, while non-normally distributed data are reported as the median with interquartile range. For normally distributed data between two groups with equal variances, unpaired *t*-tests were used; for those with unequal variances, Welch's unpaired *t*-tests were used. For normally distributed data of multiple groups, one-way analysis of variance (ANOVA) and Tukey's multiple comparison test were employed. For non-normally distributed data between two groups, the Mann-Whitney test was used, while the Kruskal-Wallis test was used for other data. For survival curves, the log-rank test was used. Statistical analysis was performed using GraphPad Prism 9.3.0 (USA).

## Results

### Elevated DCA levels inhibited intestinal epithelial cell proliferation and migration

**Figure 1:**
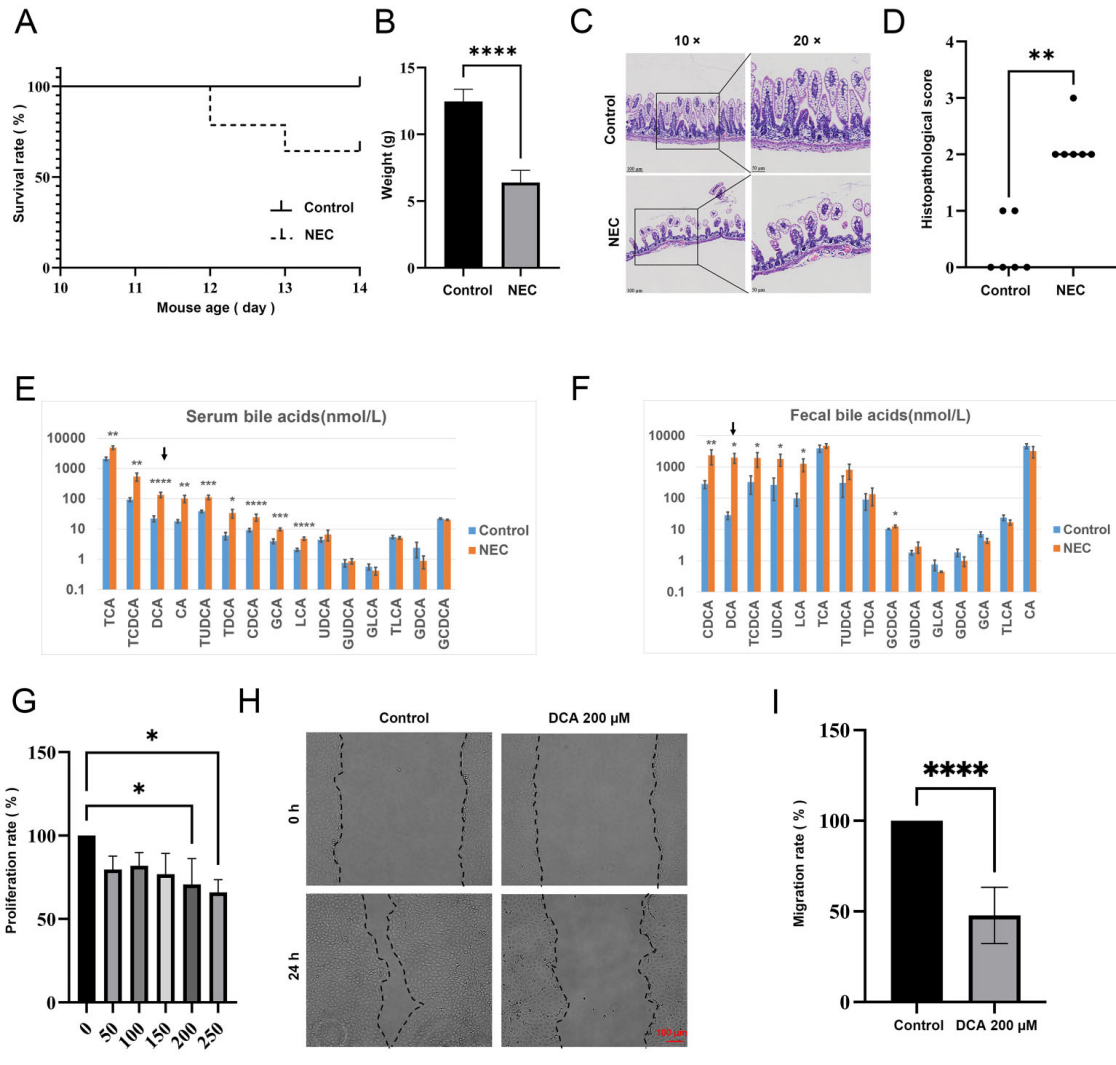
Elevated deoxycholic acid (DCA) levels in a mouse model of necrotizing. enterocolitis (NEC). **A** and **B**, Survival rate and weight of the mice on the final day of the modeling procedure (**A**, log-rank; **B**, unpaired *t*-tests). **C**, Representative photomicrographs of ileum tissue from each group (n=6; magnification ×10 and 20×, with the scale bars indicating 100 and 50 µm, respectively). **D**, Histopathological scoring of ileum tissue in mice (on a 0-4 scale; n=6; Kruskal-Wallis test). **E** and **F**, Quantification of bile acids in serum and fecal samples (n≥4; unpaired *t*-tests). **G**, IEC-6 cells treated with DCA for 24 h, and cell proliferation assessed using the CCK8 assay (n=3; unpaired *t*-tests). **H** and **I**, Migration rate of IEC-6 cells assessed by wound-healing assay after treatment with 200 µM DCA for 24 h (n=6; unpaired *t*-tests; scale bar indicates 100 µm). The data are reported as means±SD. *P<0.05, **P<0.01, ***P<0.001,****P<0.0001.

To assess the impact of NEC occurrence on bile acid levels in the mouse circulation and intestines, the levels of 15 bile acids were measured in serum and feces. Compared to those in the control group, significant alterations in the serum levels of nine bile acids, including TCA, TCDCA, DCA, CA, TUDCA, TDCA, CDCA, GCA, and LCA, were detected (Figure 1A-E). Similarly, significant changes were detected in the fecal levels of six bile acids, including CDCA, DCA, TCDCA, UDCA, LCA, and GCDCA (Figure 1F). Specifically, DCA levels were markedly elevated in both serum and feces of NEC group mice. To further understand the impact of increased DCA concentration on intestinal epithelial cell behavior, CCK8 and wound-healing experiments were conducted. Elevated DCA concentrations attenuated the proliferation capacity (Figure 1G) and migration speed of IEC-6 intestinal epithelial cells (Figure 1H and I), which suggested that the occurrence of NEC leads to elevated DCA levels in both the circulation and intestines, thereby inhibiting the proliferation and migration of intestinal epithelial cells.

### *MET* is a hub gene in the intestinal epithelial cells of NEC mice

**Figure 2:**
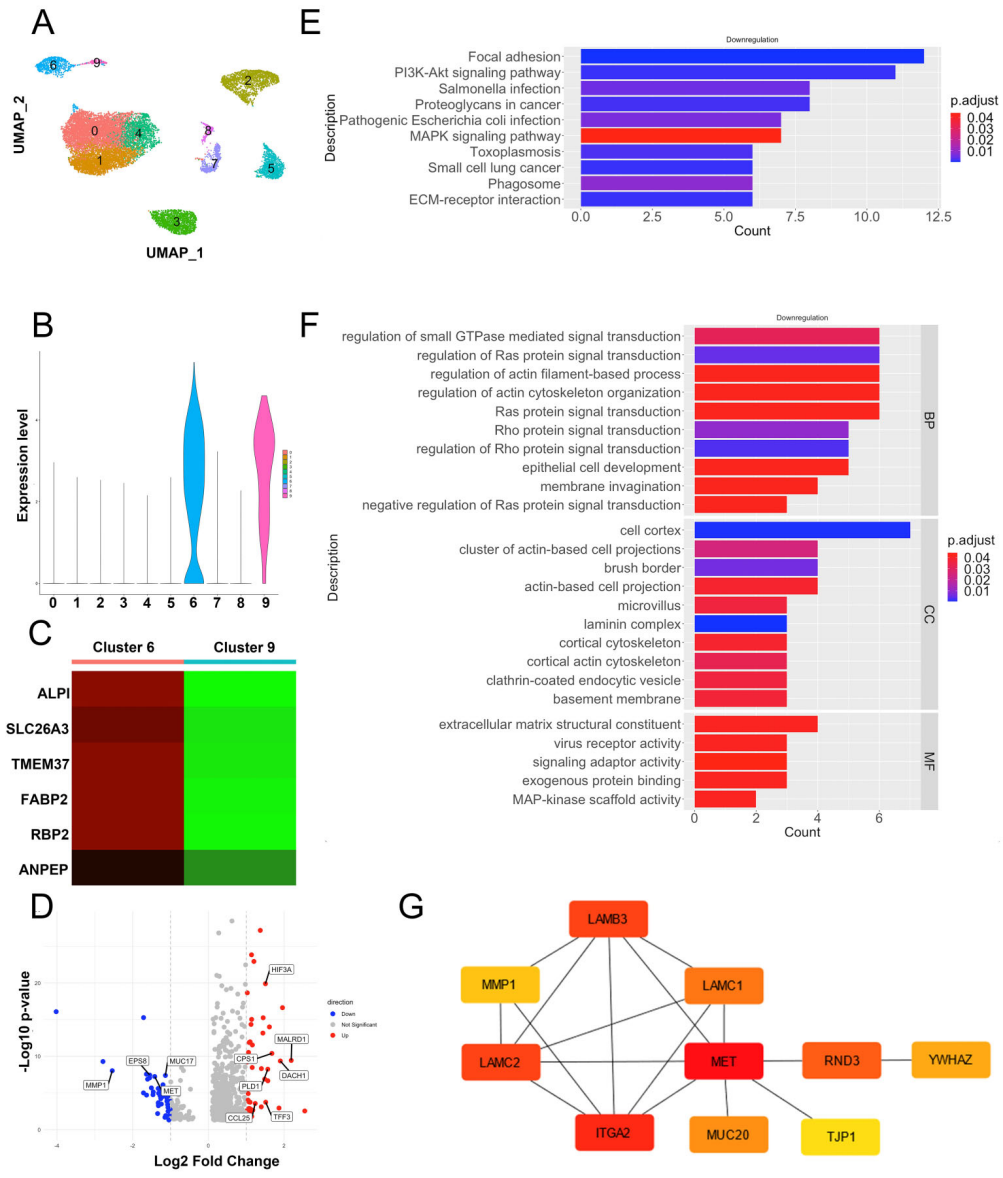
Downregulation of intestinal epithelial cell migration-associated pathways in necrotizing enterocolitis (NEC). **A**, UMAP visualization of human ileum tissue single-cell RNA-seq data (GSE178088, n=17,267). **B**, Violin plot showing the levels of the EPCAM epithelial cell marker. **C**, Heatmap displaying the expression of enterocyte markers in clusters 6 and 9. Red indicates high expression and green indicates low expression. **D**, Differential gene expression analysis in cell cluster 6, with red indicating genes upregulated and blue indicating genes downregulated with a fold change >1 in the NEC group. **E** and **F**, KEGG pathway enrichment and GO enrichment analyses of downregulated genes in cluster 6. **G**, Protein-protein interaction of downregulated genes. Red indicates a high hub gene score and yellow indicates a low hub gene score.

Intestinal epithelial cells are the first cells to encounter intestinal contents. To understand the transcriptional changes in intestinal epithelial cells under NEC conditions, the GSE178088 dataset was analyzed. After quality control, 10 clusters were obtained (Figure 2A). Cluster 6 and cluster 9 exhibited high expression of the EPCAM epithelial cell marker (Figure 2B), suggesting that cluster 6 represents a subgroup of intestinal epithelial cells (Figure 2C).

Differential gene expression analysis revealed 53 upregulated genes and 52 downregulated genes under NEC conditions (Figure 2D). The upregulated genes included inflammation-related genes in the TLR4 pathway, such as *CCL25* and *PLD1*, as well as hypoxia-inducible factor HIF-3α and the MALRD1 bile acid synthesis negative regulator. Conversely, the downregulated genes included genes involved in cell proliferation, motility, and intestinal epithelial protection, such as *MUC17*, *MET*, and *MMP1*.

These findings suggested that the proliferative and motility functions of intestinal epithelial cells may be suppressed under NEC conditions. Pathway analysis of the downregulated genes revealed a reduction in pathways associated with cell-cell and cell-matrix interactions involved in cell migration (Figure 2E and F). Through PPI network construction and hub gene calculation, *MET* was identified as one of the top-ranked downregulated genes (Figure 2G).

In summary, the occurrence of NEC creates an environment characterized by inflammation, hypoxia, and bile acid imbalance in intestinal epithelial cells. Additionally, the migratory capacity of epithelial cells may be inhibited, with *MET* potentially serving as a hub gene in this process.

### DCA inhibited the proliferation and migration of IEC-6 intestinal epithelial cells by downregulating MET

**Figure 3:**
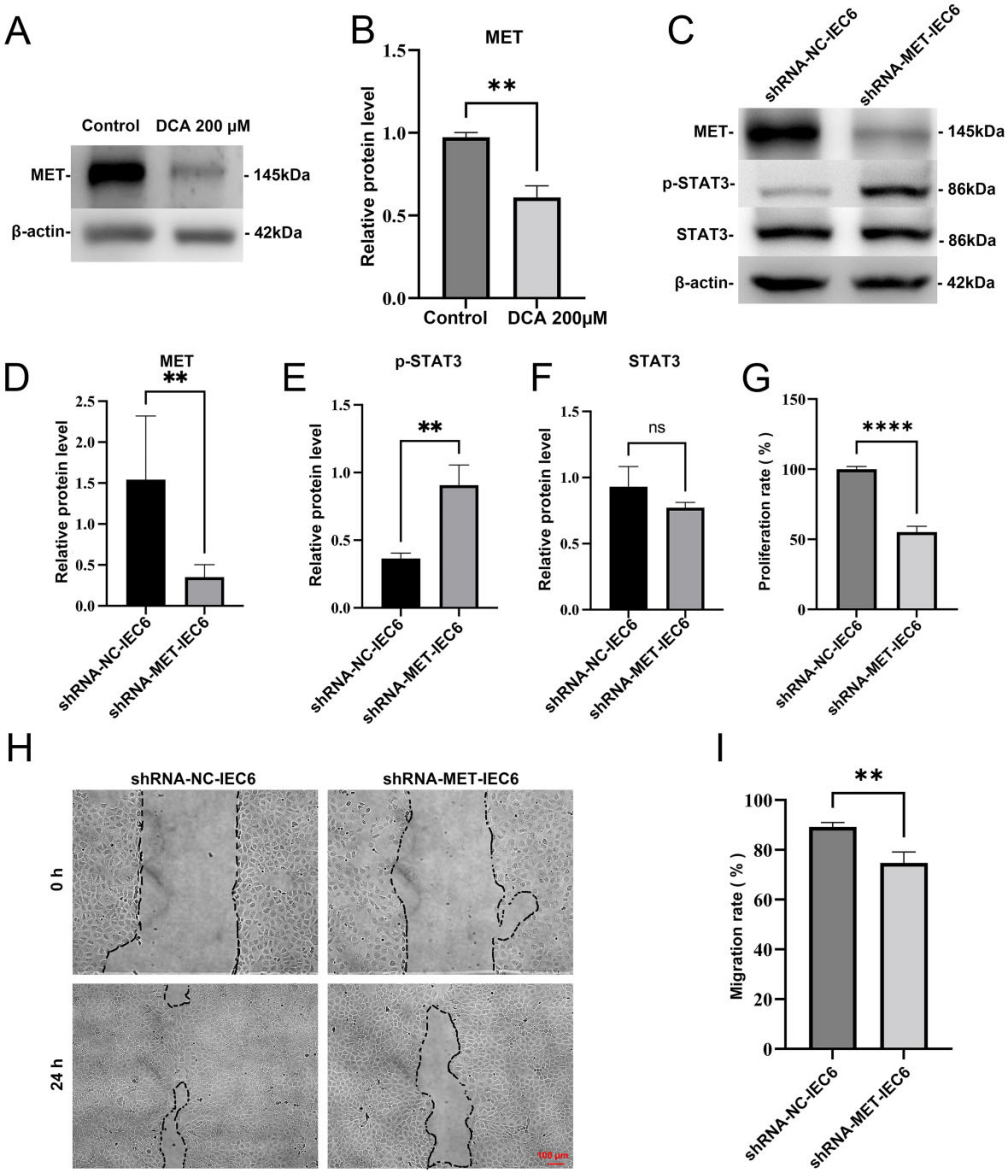
Deoxycholic acid (DCA) suppressed the proliferation and migration of intestinal epithelial cells via mesenchymal-epithelial transition (MET) factor inhibition. **A** and **B**, IEC-6 cells were treated with 200 µM DCA for 24 h, followed by western blot analysis to detect MET expression. **C-F**, The expression levels of MET, p-STAT3, and STAT3 were measured in MET-deficient IEC-6 cells by western blot analysis. **G**, Proliferation rate of control and MET-deficient IEC-6 cells assessed by a CCK8 assay. **H** and **I**, The migration capacity of MET-deficient IEC-6 cells was evaluated using a wound-healing assay. Scale bar indicates 100 µm. The data are reported as means±SD (n=3). **P<0.01 and ****P<0.0001, unpaired *t*-tests. ns: non-significant.

The present results demonstrated that DCA suppressed the proliferation and migration of IEC-6 intestinal epithelial cells and that MET may be a key gene associated with the reduced migratory capacity of intestinal epithelial cells in NEC. To investigate the relationship between DCA and MET, the expression of MET was evaluated in DCA-treated IEC-6 cells. DCA significantly inhibited the protein expression of MET in intestinal epithelial cells (Figure 3A and B). Moreover, MET knockdown IEC-6 cells (Figure 3C and D) revealed that MET deficiency significantly inhibited the proliferation (Figure 3G) and migration abilities of IEC-6 cells (Figure 3H and I). Additionally, the phosphorylation of STAT3 was significantly increased in MET-deficient cells (Figure 3E and F). These findings suggested that the inhibition of intestinal epithelial cell proliferation and migration by DCA was achieved through downregulation of MET expression, which increased phosphorylation of STAT3 in intestinal epithelial cells.

### DCA reduced intestinal MET levels in NEC mice, increased p-STAT3 nuclear translocation, and aggravated NEC injury

**Figure 4:**
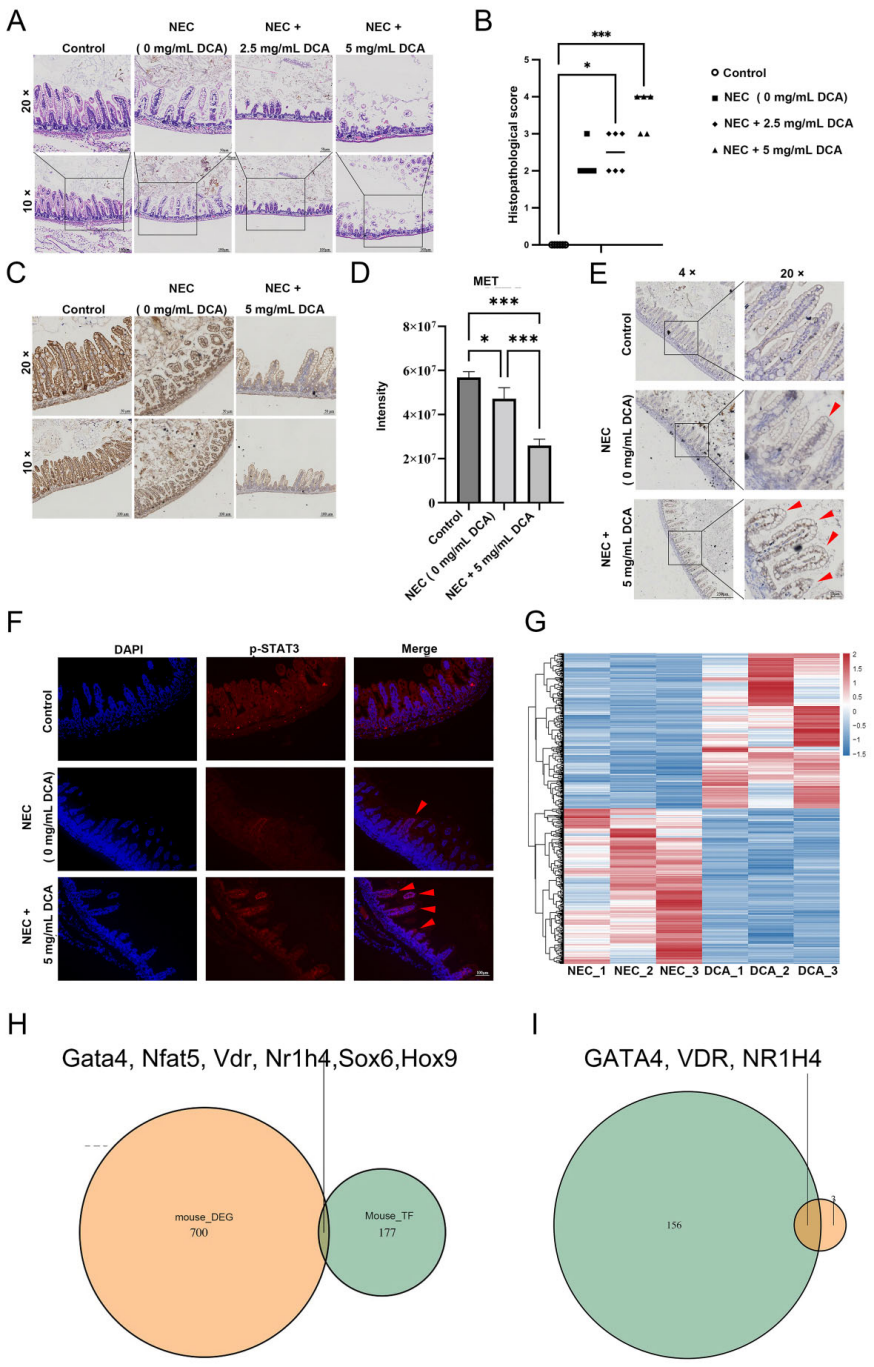
Deoxycholic acid (DCA) exacerbates intestinal damage in necrotizing enterocolitis (NEC) mice. **A** and **B**, Representative photomicrographs of ileum tissue from each group (magnification 10× and 20×, with scale bars indicating 100 and 50 µm, respectively; n=5-6; Kruskal-Wallis test). **C** and **D**, DCA inhibited mesenchymal-epithelial transition factor (MET) expression in NEC mice, as evidenced by immunohistochemistry (IHC) analysis of ileum tissue from each group (n=3). Scale bars indicate 50 and 100 μm, respectively. The data are reported as means±SD. *P<0.05 and ***P<0.001 (unpaired *t*-tests). **E** and **F**, Immunohistochemistry (IHC) and immunofluorescence (IF) detection of p-STAT3 levels and nuclear translocation in the ileal tissues of each group, with red arrows indicating co-localization of p-STAT3 with the cell nucleus. The magnifications are 4× (IHC), 10× (IF), and 20× (IHC), with scale bars indicating 250 µm (IHC), 100 µm (IF), and 50 µm (IHC), respectively. **G**, Heatmap depicting differential gene expression between the NEC and NEC + 5 mg/mL DCA groups. **H**, Intersection analysis between differentially expressed genes (DEGs) in mice (orange) and predicted transcription factors (TF) of mouse MET (green). **I**, Intersection analysis between predicted TF of human MET (green) and predicted mouse transcription factors affected by DCA in NEC (orange).

To investigate the effects and mechanisms of DCA in NEC mice, graded doses of DCA were administered during NEC modeling. Histopathological examination revealed a progressive worsening of intestinal injury in NEC mice with increasing DCA concentrations (Figure 4A and B). The DCA groups had higher pathological scores compared to the control group, and the 5 μg/μL group had significantly greater pathological scores compared to the NEC group. To investigate the additional damage caused by DCA in NEC, the expression of MET was assessed in the intestinal tissues of the control, NEC, and 5 μg/μL DCA groups (Figure 4C and D). Immunohistochemistry (IHC) revealed a decrease in MET expression in both the NEC and 5 μg/μL DCA groups compared to the control group, with a further decrease in MET expression observed in the 5 μg/μL DCA group compared to the NEC group (Figure 4C and D). IHC and immunofluorescence (IF) analyses revealed increased nuclear localization of p-STAT3 in the DCA-treated groups (Figure 4E and F). These findings suggested that DCA accumulation in NEC downregulated intestinal MET expression levels, thereby exacerbating intestinal injury in NEC mice and promoting the nuclear translocation of p-STAT3.

### GATA4, NR1H4, and VDR are candidate transcription factors regulating MET in response to DCA

To elucidate how DCA regulates MET, the upstream regulatory factors of MET that may be influenced by DCA were predicted. First, whole-transcriptome sequencing was performed to detect transcriptional changes in NEC mice treated with DCA. DCA had a significant impact on transcription, with 352 upregulated genes and 354 downregulated genes identified (Figure 4G). Subsequently, 183 potential mouse MET transcription factors were identified from the JASPAR database. Intersection analysis identified six predicted transcription factors affected by DCA (Figure 4H). To assess whether these factors are associated with the occurrence of NEC, an intersection analysis of the differentially expressed genes in the transcriptome of patients with NEC and the JASPAR-predicted results of human MET was performed, which revealed 159 potential transcription factors influencing MET in NEC pathogenesis. By comparing the predicted transcription factors affected by DCA with those associated with NEC, three transcription factors were identified (Figure 4I). These findings suggested that GATA4, VDL, and NR1H4 may play a role in the DCA-mediated regulation of MET in NEC.

## Discussion

**Figure 5:**
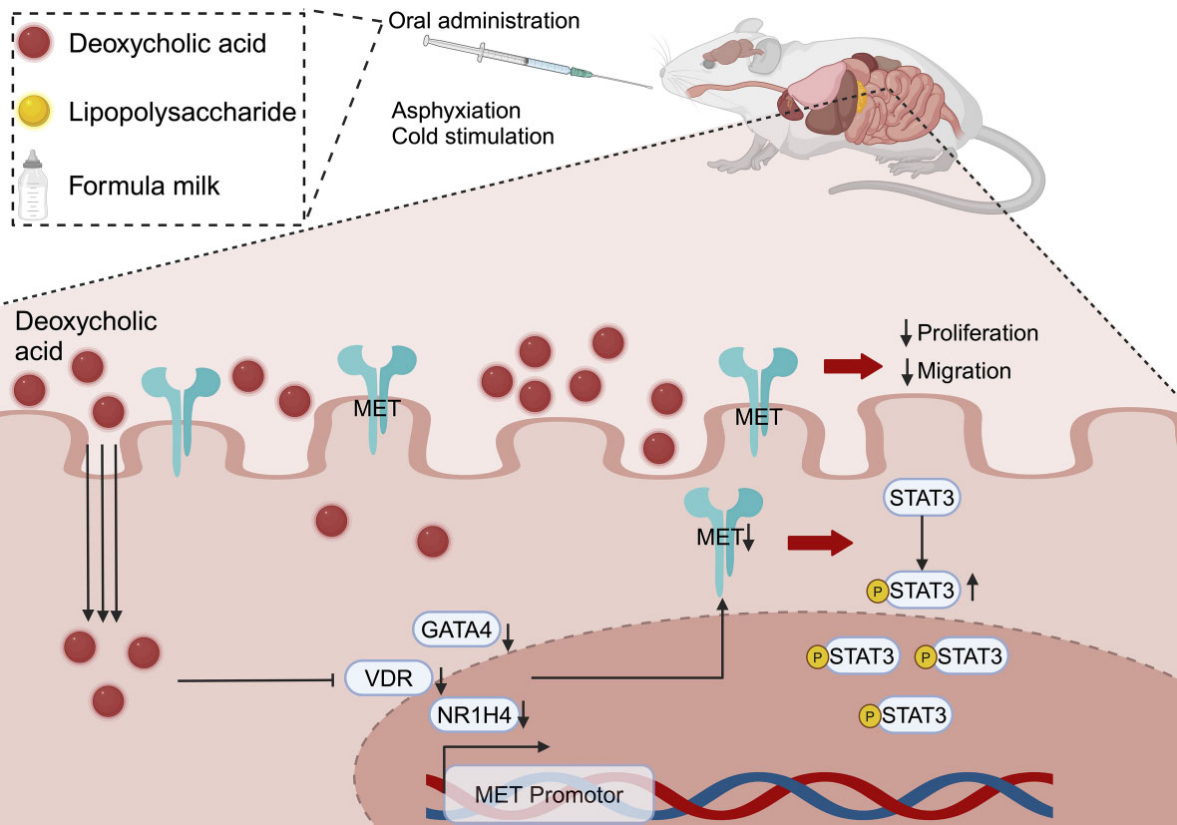
A schematic illustration depicting how deoxycholic acid exacerbates necrotizing enterocolitis (NEC) by downregulating mesenchymal-epithelial transition (MET) factor.

Abnormalities in bile acid levels have been implicated in NEC, but research progress in this area has been slow (6). The present study identified changes in bile acid composition induced by NEC occurrence using a mouse model. Significant alterations were observed in the levels of DCA, both in blood and fecal samples. Analysis of single-cell transcriptome sequencing data and *in vivo* and *in vitro* experiments demonstrated that DCA downregulated MET, which in turn affected the proliferation and migration of intestinal epithelial cells (Figure 5). These functions are considered crucial components of intestinal injury repair (25). Thus, abnormalities in DCA metabolism exacerbate NEC damage by impairing intestinal injury repair mechanisms.

The role of impaired intestinal injury repair in intestinal inflammation has been reported to lead to the downregulation of tight junction proteins in intestinal epithelial cells, resulting in intestinal barrier damage and bacterial translocation, which is considered a critical part of NEC pathogenesis (2,26). Studies have shown that the removal of intestinal bile acids alleviates NEC, but the specific role of which bile acid subtype is involved remains unclear (27). Our previous findings have demonstrated that LCA inhibits intestinal epithelial cell proliferation, exacerbating NEC damage (9). Thus, the abnormalities in bile acid metabolism that lead to impaired intestinal injury repair function are significantly involved in NEC development. The present study demonstrated that DCA inhibited the proliferation and migration of intestinal epithelial cells and exacerbated NEC-induced intestinal injury with increasing DCA concentrations. Similarly, a previous study has indicated that a high concentration of DCA leads to NEC-like ileal damage, while a low concentration of DCA does not have this effect. Notably, gavage with 8.291 mg/mL (20 mM) DCA without NEC modeling does not cause significant ileal damage, whereas the use of 5 mg/mL DCA for NEC modeling in the present study resulted in a significant increase in ileal damage. These results suggested that the effect of DCA on NEC may vary. Further research on the sensitivity of NEC to DCA is warranted in the future. DCA also has varying effects on colorectal cancer cells depending on its concentration, promoting cell proliferation at low concentrations and inducing apoptosis at high concentrations (28). DCA exacerbates intestinal inflammation by increasing interleukin (IL)-1β expression to activate CD3^+^ and CD4^+^ cells *in vivo* (29). The trends observed in the present study are consistent with those findings. Additionally, our previous study has demonstrated that melatonin alleviates intestinal injury in NEC by improving bile acid metabolism and reducing DCA levels (18), suggesting that DCA plays a role in exacerbating damage in NEC and may serve as a potential target for NEC treatment.

MET is a receptor tyrosine kinase that is expressed in most epithelial cells. MET is involved in several biological processes, including wound healing, proliferation, and migration (19,30,31). MET has also been reported to participate in the repair of intestinal injury (21). The present study revealed that DCA inhibited the expression of MET in intestinal epithelial cells and the intestines of NEC model mice. By binding to its receptor, DCA regulates gene expression (32). Therefore, the present study predicted transcription factors affected by DCA that are capable of binding to MET, which identified the GATA4, VDR, and NR1H4 candidate transcription factors. The accumulation of DCA in the ileum inhibits the expression of FXR and related proteins (33). Similarly, the present transcriptome sequencing results predicted FXR as a candidate transcription factor. The present findings suggested that MET serves as the target site of DCA in NEC, and they provided new insights into the mechanism by which DCA regulates MET expression.

The activation of Toll-like receptor 4 (TLR4) in intestinal epithelial cells (IECs) has been suggested to lead to the recruitment of immature lymphocytes and induce differentiation toward inflammatory Th17 cells via the STAT3 pathway in NEC (34). We have previously reported the therapeutic effect of blocking the IL-6/STAT3 pathway using IL-6R antibodies to reverse the imbalance in the Treg/Th17 ratio in NEC (35). However, the role of STAT3 activation in intestinal epithelial cells is different. Reports have suggested that STAT3 regulates intestinal homeostasis, and mice with specific deletion of STAT3 in intestinal epithelial cells are more susceptible to experimental colitis, with downregulation of injury repair-related pathways (36,37). It has been reported that activated STAT3 interacts with the *MET* gene promoter region, leading to the upregulation of its transcription (38). The present results indicated that *MET* knockdown leads to increased phosphorylation of STAT3 in IEC-6 cells and that DCA promotes the nuclear localization of p-STAT3 in the intestinal epithelial cells of NEC mice. Similarly, in gastric epithelial cells, DCA has been observed to mediate adverse outcomes of gastric inflammation and intestinal metaplasia, as well as to lead to increased STAT phosphorylation and nuclear translocation (39). Previous research has shown that the use of MET inhibitors upregulates STAT3 activation via the JAK/STAT3 pathway (40), which is consistent with the present results. Therefore, the present data suggested that the downregulation of MET by DCA leads to the nuclear translocation of p-STAT3 in intestinal epithelial cells. Due to the lack of specific techniques for reducing DCA levels in the intestinal lumen in the animal model, we were unable to observe the therapeutic effect of removing DCA in NEC, thus limiting the generalizability of the present results. In the future, it will be necessary to explore techniques for specifically reducing DCA levels in the intestinal lumen to study the role of bile acids in NEC.

In summary, the present findings suggested that the accumulation of DCA in the intestinal lumen exacerbates intestinal injury in NEC mice via a mechanism related to the inhibition of intestinal epithelial cell proliferation and migration caused by DCA-mediated downregulation of MET expression. These results further support the mechanisms by which DCA affects NEC, providing new evidence for the pathogenesis of NEC.
